# Post-glucose-load urinary C-peptide and glucose concentration obtained during OGTT do not affect oral minimal model-based plasma indices

**DOI:** 10.1007/s12020-015-0765-9

**Published:** 2015-11-02

**Authors:** Sjaam Jainandunsing, J. L. Darcos Wattimena, Trinet Rietveld, Joram N. I. van Miert, Eric J. G. Sijbrands, Felix W. M. de Rooij

**Affiliations:** Department of Internal Medicine, Erasmus MC - University Medical Center Rotterdam, Room Na-512, PO-box 2040, 3000 CA Rotterdam, The Netherlands

**Keywords:** Type 2 diabetes, OGTT, Oral minimal model, C-peptide, Glucose, South Asian

## Abstract

The purpose of this study was to investigate how renal loss of both C-peptide and glucose during oral glucose tolerance test (OGTT) relate to and affect plasma-derived oral minimal model (OMM) indices. All individuals were recruited during family screening between August 2007 and January 2011 and underwent a 3.5-h OGTT, collecting nine plasma samples and urine during OGTT. We obtained the following three subgroups: normoglycemic, at risk, and T2D. We recruited South Asian and Caucasian families, and we report separate analyses if differences occurred. Plasma glucose, insulin, and C-peptide concentrations were analyzed as AUCs during OGTT, OMM estimate of renal C-peptide secretion, and OMM beta-cell and insulin sensitivity indices were calculated to obtain disposition indices. Post-glucose load glucose and C-peptide in urine were measured and related to plasma-based indices. Urinary glucose corresponded well with plasma glucose AUC (Cau *r* = 0.64, *P* < 0.01; SA *r* = 0.69, *P* < 0.01), *S*_I_ (Cau *r* = −0.51, *P* < 0.01; SA *r* = −0.41, *P* < 0.01), *Φ*_dynamic_ (Cau *r* = −0.41, *P* < 0.01; SA *r* = −0.57, *P* < 0.01), and *Φ*_oral_ (Cau *r* = −0.61, *P* < 0.01; SA *r* = −0.73, *P* < 0.01). Urinary C-peptide corresponded well to plasma C-peptide AUC (Cau *r* = 0.45, *P* < 0.01; SA *r* = 0.33, *P* < 0.05) and OMM estimate of renal C-peptide secretion (*r* = 0.42, *P* < 0.01). In general, glucose excretion plasma threshold for the presence of glucose in urine was ~10–10.5 mmol L^−1^ in non-T2D individuals, but not measurable in T2D individuals. Renal glucose secretion during OGTT did not influence OMM indices in general nor in T2D patients (renal clearance range 0–2.1 %, with median 0.2 % of plasma glucose AUC). C-indices of urinary glucose to detect various stages of glucose intolerance were excellent (Cau 0.83–0.98; SA 0.75–0.89). The limited role of renal glucose secretion validates the neglecting of urinary glucose secretion in kinetic models of glucose homeostasis using plasma glucose concentrations. Both C-peptide and glucose in urine collected during OGTT might be used as non-invasive measures for endogenous insulin secretion and glucose tolerance state.

## Introduction

Mathematical approaches based on compartmental pharmacokinetic/pharmacodynamic (PK/PD) principles are used to describe the biphasic glucose–insulin system in oral function tests [1, 2], with the oral minimal model (OMM) as one of the most widely accepted approaches [3]. However, the contribution of renal clearance of endogenous glucose, insulin, and C-peptide during oral glucose tolerance test (OGTT) in various stages of glucose tolerance remains largely unclear. As renal extraction of insulin is negligible [4], we focused on the relationship between plasma and urine concentrations of both C-peptide and glucose, collected during OGTT in the post-glucose load phase. This was performed in families to obtain groups with different risk for T2D, and we recruited families of South Asian and Caucasian origin to enable generalization of our findings. Especially, South Asians with T2D may be at high risk for chronic kidney disease [5]. We questioned to which degree C-peptide and glucose excretion in urine influence OGTT-based plasma indices. Moreover, we compared the OMM-derived estimates of renal C-peptide excretion with actual urinary C-peptide concentration. In addition, renal loss of glucose is not taken into consideration in OMM, and the extent to which renal clearance might require correction of plasma-derived OMM calculations is unknown.

## Methods

### Subjects and anthropometric data

Patients were recruited from South Asian and Caucasian families with high risk of T2D after family screening from the Outpatient Clinic of the Erasmus Medical University Centre as described previously [6]. The first-degree relatives of patients with T2D attending our Clinic (index cases), who did not have T2D were recruited from 36 South Asian families and 24 Caucasian families, with 2 generations taken into account. Data were obtained from 57 (M29 F28) South Asians and 64 (M24 F40) Caucasians who all underwent an OGTT. Index cases were on metformin use only and had at least one sibling with T2D. Informed written consent to the study was obtained from all participants. The study protocol was approved by the Erasmus University Medical Center Medical Ethics Review Board. All procedures followed were in accordance with the ethical standards of the responsible committee on human experimentation (institutional and national) and with the Helsinki Declaration of 1975, as revised in 2008.

### OGTT

Venous blood was drawn via an intravenous canula, at time-points 60 and 15 min before 75 g glucose load and 15, 30, 45, 60, 90, 120, 150, 180, and 210 min after glucose load to measure glucose, insulin, and C-peptide concentrations. The WHO criteria for the OGTT were used to define normal glucose tolerance (NGT), impaired fasting glucose and/or impaired glucose tolerance (IFG/IGT), or Type 2 diabetes (T2D) status among subjects. After emptying their bladder prior to glucose load, urine was collected until 210 min after glucose load. As there were no significant differences between baseline values of plasma glucose, insulin, or C-peptide obtained at −60 min or −15 min, and as −60 min was sampled before our study subjects had emptied their bladder prior to glucose load, we chose −60 min as the representative baseline value. In 7 of 18 Caucasians with T2D, urine was not collected, because it was not included in our protocol at that time. As this group did not significantly differ from the remaining group, we used data of *n* = 18 for all plasma indices, but data of *n* = 11 for all analyses with urinary glucose and C-peptide measurements in Caucasians.

### Immunoassay

Plasma and urine glucose was measured by a hexokinase-based method (Gluco-quant; Roche Diagnostics, Mannheim, Germany). Plasma and urine C-peptide, and plasma insulin were measured separately by a competitive chemiluminescent immunoassay, supplied by Euro/DPC. The assay was performed on a DPC Immulite 2000 analyzer (Euro/DPC) according to the manufacturer’s recommended protocol. Serum creatinine was measured with an enzymatic procedure based on creatinine conversion with the Creatinine Plus assay on a Roche/Hitachi analyzer. Urine creatinine was measured based on the Jaffe alkaline picrate method.

### Calculations for OMM

**Fig. 1:**
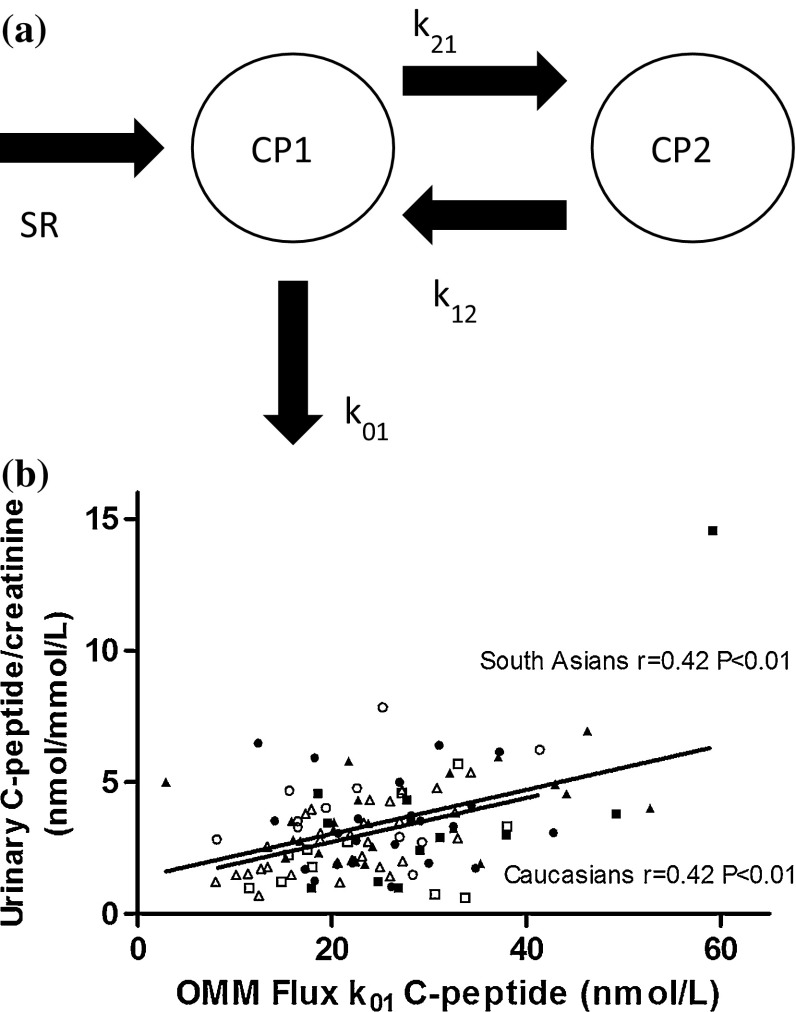
**a** Two-compartment model of C-peptide kinetics that is integrated in oral minimal model (OMM). SR is prehepatic insulin secretion rate, based on C-peptide curve. CP1 represents the amount of C-peptide in central compartment and CP2 the amount of C-peptide in peripheral compartment. *k*_21_ and *k*_12_ are C-peptide transfer rates between CP1 and CP2; *k*_01_ describes metabolization of C-peptide from CP1. In this study, plasma AUC of CP1*k_01_, or OMM flux *k*_01_, is related to actual measured C-peptide concentration in urine during OGTT. Adapted from van Cauter et al. [1]. **b** Correlation between C-peptide in urine and OMM flux *k*_01_. NGT (*triangle*), IFG/IGT (*square*), and T2D (*circle*) subgroups for South Asian (*closed*) and Caucasian (*open*) families

The OMM was used to describe the plasma glucose, insulin, and C-peptide concentrations after oral glucose stimulus [7]. With C-peptide minimal model, we assessed parameters for beta-cell function: basal responsivity of beta-cells due to basal glucose potentiation *Φ*_basal_(min^−1^), static responsivity of beta-cells due to glucose potentiation *Φ*_static_ (10^−9^ min^−1^), dynamic responsivity of beta-cells due to glucose potentiation *Φ*_dynamic_ (10^−9^), total responsivity of beta-cells due to glucose potentiation *Φ*_oral_ (10^−9^ min^−1^), and delay in response to glucose potentiation *T* (min). With glucose minimal model, we assessed parameters for insulin sensitivity, insulin sensitivity *S*_I_ (10^−5^ dL kg^−1^ min^−1^ per pM). Parameters from both models were multiplied with each other for calculation of disposition indices (DI), which are beta-cell function measures corrected for insulin sensitivity: DI_basal_ *=* *Φ*_basal_*S_I_, DI_static_ *=* *Φ*_static_*S_I_, DI_dynamic_ *=* *Φ*_dynamic_*S_I_, and DI_oral_ *=* *Φ*_oral_*S_I_. Parameters of OMM were estimated with SAAM2 software [8]. Incremental plasma AUC of C-peptide and glucose within a given time period was calculated according to trapezoidal rule, with subtraction of basal concentration. For urinary glucose, we estimated plasma glucose threshold (when exceeded glucose in urine is present) separately among both our T2D and non-T2D (NGT + IFG/IGT) groups; we calculated plasma glucose AUCs from 8.5 to 11.5 mmol L^−1^ with an interval of 0.5 mmol L^−1^ to detect the most suitable plasma glucose threshold. Stepwise exclusion was performed of T2D and non-T2D individuals, based on whether their plasma glucose AUC was above a given threshold or not. Also, to determine the relative renal loss of glucose from total plasma glucose AUC, renal clearance of glucose was calculated with absolute amount of urinary glucose/total plasma glucose AUC. For comparison with urinary C-peptide, we used AUC from flux *k*_01_ (Fig. 1) from OMM, representing irreversibly metabolized C-peptide from central compartment. Estimated glomerular filtration rate (eGFR) was estimated with the modification of diet in renal disease (MDRD) formula [9, 10].

### Statistical analyses

#### Data analysis

Data are expressed as mean ± SEM, or indicated otherwise. Comparisons within the subgroups of the ethnicities were done with ANOVA, with the other two subgroups of same ethnicity and with the corresponding other ethnic subgroup. Differences were considered statistically significant when the two-sided *P* value was <0.0125. Urinary glucose and C-peptide were correlated with plasma indices, with Spearman correlation within each ethnicity, with significance at *P* value <0.05. For urinary glucose and C-peptide concentrations, AUC of receiver-operated characteristics (ROC) curves (concordance indices or c-indices), adjusted for family ties by binary logistic regression analysis [11], were calculated to detect IFG/IGT and/or T2D status. All statistical tests were conducted with the use of SPSS, version 15.0, for Windows (SPSS Inc., Chicago, IL, USA).

## Results

### Baseline characteristics

**Fig. 2:**
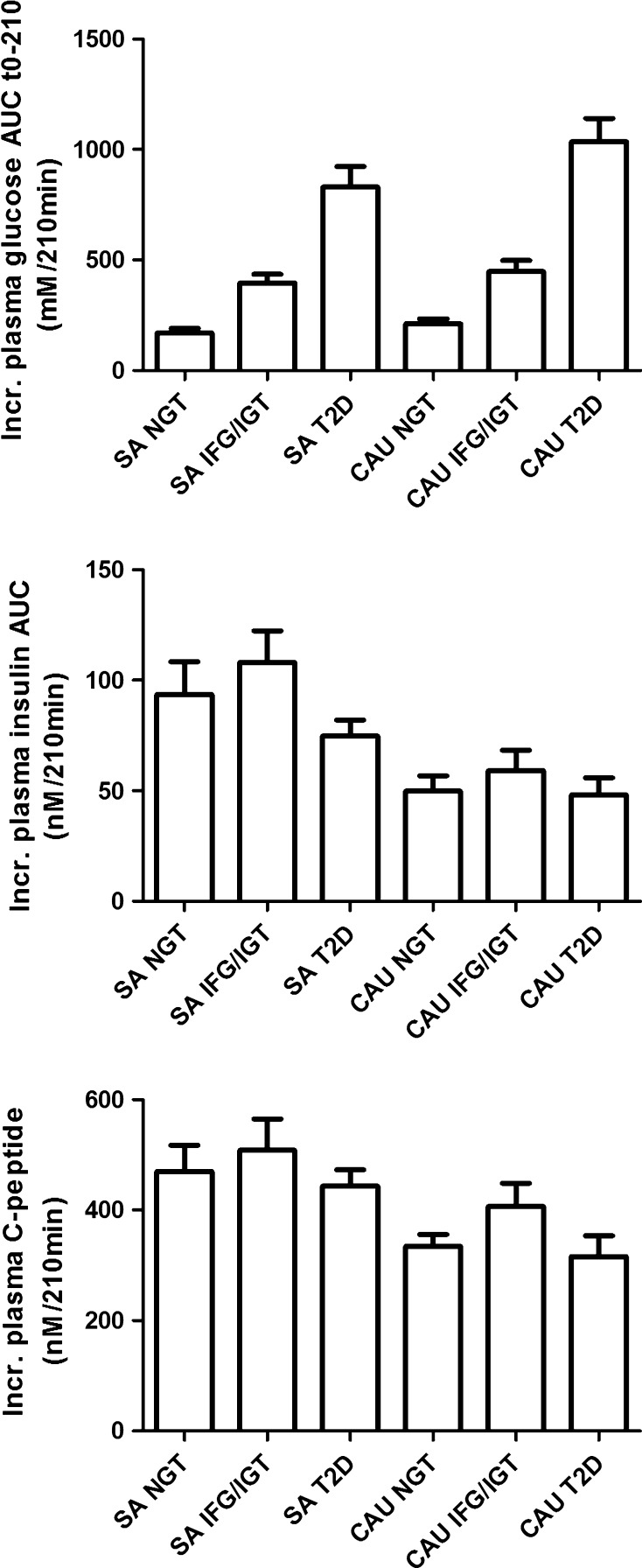
Incremental plasma AUC of glucose, insulin and C-peptide during 210 min OGTT (mean ± SEM) among WHO OGTT subgroups with normal glucose tolerance (NGT), impaired fasting glucose and/or impaired glucose tolerance (IFG/IGT) and Type 2 diabetes (T2D) from South Asian (SA) or Caucasian (Cau) origin. Incr. plasma glucose AUC; SA NGT versus T2D SA *P* < 0.001, SA IFG/IGT versus T2D *P* < 0.001, Cau NGT versus Cau T2D *P* < 0.001, CauIFG/IGT versus Cau T2D *P* < 0.001. Incr. plasma insulin AUC; SA NGT versus Cau NGT *P* < 0.01. Please note different scale in Y-axis for insulin and C-peptide.

Baseline characteristics are shown in Table 1 and incremental plasma AUC of primary data glucose, insulin, and C-peptide during OGTT in Fig. 2. South Asian T2D individuals were younger (*P* = 0.004), shorter (*P* < 0.001), and weighed less (*P* = 0.005) than Caucasian T2D individuals. W/H increased from NGT to T2D in Caucasians (*P* = 0.003), but not in South Asians. In both ethnicities, incremental plasma glucose AUCs from NGT or IFG/IGT were significantly lower than T2D (*P* < 0.001). In both ethnicities, no significant differences were found in incremental plasma insulin AUC between NGT, IFG/IGT, and T2D; however, a difference was found between South Asian NGT and Caucasian NGT (*P* < 0.01). In both ethnicities, no significant differences were found in incremental plasma C-peptide AUC between NGT, IFG/IGT, and T2D. In both ethnicities, no significant differences were found in *Φ*_basal_ between NGT, IFG/IGT, and T2D, whereas, *Φ*_dynamic_, *Φ*_static_, and *Φ*_total_ decreased with increasing glucose intolerance. No significant differences were found in delay *T*. With OMM, we observed a decrease in insulin sensitivity in both ethnicities with increasing glucose intolerance (*P* < 0.001); however, differences were lower among the South Asian subgroups. All disposition indices decreased with increasing glucose intolerance. Overall, South Asian NGT and IFG/IGT demonstrated lower DI indices when compared to Caucasian NGT, whereas those of South Asian T2D were higher than their Caucasian counterparts. In both ethnicities, glucose in urine increased with increasing glucose intolerance; both creatinine-unadjusted and adjusted urinary glucose in T2D subgroup were significantly higher when compared to NGT or IFG/IGT subgroups (*P* < 0.001). In Caucasians, creatinine adjusted C-peptide was higher in T2D when compared to NGT and IFG/IGT combined (*T* test *P* = 0.005); no differences were found among South Asians.

**Table 1:** Clinical characteristics in persons with NGT, IGT and/or IFG, and type 2 diabetes

	South Asian			Caucasian		
	NGT	IFG/IGT	T2D	NGT	IFG/IGT	T2D
*n*	22	12	23	34	12	18
Sex (male/female), *n*% (male)	10/12 (45.5)	8/4 (66.7)	11/12 (47.8)	11/23 (32.4)	4/8 (33.3)	9/9 (50.0)
Age (years)	39.6 ± 11.6^a^	46.3 ± 8.8	52.3 ± 8.8^b,c^	38.9 ± 9.4^b^	44.5 ± 11.4^b^	63.2 ± 7.6^a,d,e^
Weight (kg)	78.7 ± 13.8	78.7 ± 14.5	74.4 ± 12.4^b^	81.1 ± 15.7	94.1 ± 30.4	90.5 ± 15.0^a^
Length (m)	1.69 ± 0.1	1.67 ± 0.1	1.61 ± 0.1^b^	1.75 ± 0.1	1.75 ± 0.1	1.76 ± 0.1^a^
BMI (kg m^−2^)	27.6 ± 4.1	27.9 ± 2.9	28.6 ± 4.1	26.3 ± 4.1	30.4 ± 8.5	29.3 ± 4.9
Waist (cm)	94 ± 10	98 ± 13	97 ± 11	91 ± 15^b^	105 ± 20	105 ± 14^d^
Hip (cm)	105 ± 5	103 ± 6	104 ± 9	108 ± 8	116 ± 20	112 ± 10
W/H ratio	0.90 ± 0.07	0.95 ± 0.10	0.93 ± 0.08	0.84 ± 0.09^b^	0.90 ± 0.07	0.94 ± 0.08^d^
eGFR MDRD (mL min^−1^)	103.2 ± 5.0	112.9 ± 6.8	102.5 ± 5.7	101.8 ± 3.2	104.1 ± 7.6	94.2 ± 3.9
*Φ*_basal_ (min^−1^)	0.50 ± 0.05	0.54 ± 0.07	0.42 ± 0.02	0.36 ± 0.02	0.36 ± 0.03	0.38 ± 0.05
*Φ*_dynamic_ (10^−9^)	365.36 ± 41.89^a^	228.19 ± 62.60	114.52 ± 36.50^c^	252.55 ± 28.55^b^	140.48 ± 40.55	94.46 ± 15.10^d^
*Φ*_static_ (10^−9^ min^−1^)	21.28 ± 1.17^a,f^	12.64 ± 1.35^c^	8.33 ± 1.24^c^	18.34 ± 1.71^b^	11.97 ± 1.46	4.19 ± 0.52^d^
*Φ*_oral_ (10^−9^ min^−1^)	24.96 ± 1.38^a,f^	14.66 ± 1.49^c^	9.43 ± 1.58^c^	21.37 ± 1.89^b^	13.88 ± 1.86	4.92 ± 0.61^d^
T (min)	13.15 ± 2.61	8.93 ± 1.05	14.84 ± 2.06	10.35 ± 1.06	11.69 ± 1.74	13.86 ± 2.50
*S*_I_ (10^−5^ dL kg^−1^ min^−1^ per pM)	15.60 ± 2.60^a^	8.30 ± 1.80	7.60 ± 1.40^c^	26.70 ± 3.70^b^	12.90 ± 3.60	5.80 ± 1.50^d^
DI_basal_ (10^−5^ dL kg^−1^ min^−2^ per pM)	6.40 ± 0.70^a^	3.90 ± 0.60	3.10 ± 0.60^c^	8.70 ± 1.20^b^	4.30 ± 1.20	1.60 ± 0.30^d^
DI_dynamic_ (10^−14^ dL kg^−1^ min^−1^ per pM)	5263.30 ± 902.50^a^	2116.00 ± 888.30	739.20 ± 205.20^c^	6389.70 ± 1373.20^b^	1999.90 ± 631.50	390.70 ± 115.10^d^
DI_static_ (10^−14^ dL kg^−1^ min^−2^ per pM)	323.40 ± 52.80^a,f^	111.60 ± 32.90^c^	60.50 ± 11.60^c^	558.90 ± 147.60^b^	144.30 ± 43.40	23.40 ± 7.10^d^
DI_oral_ (10^−14^ dL kg^−1^ min^−2^ per pM)	379.90 ± 62.40^a,f^	130.30 ± 38.10^c^	67.70 ± 13.00^c^	637.10 ± 164.60^b^	165.10 ± 49.10	26.80 ± 8.00^d^
Glucose urine (mmol L^−1^)	0.31 ± 0.24^a^	2.13 ± 1.29^a^	24.99 ± 7.02^c,f^	0.19 ± 0.06^b^	4.55 ± 3.37^b^	32.68 ± 6.57^A,d,e^
Glucose/creatinine ratio urine	0.04 ± 0.02^a^	0.33 ± 0.20^a^	5.60 ± 1.67^c,f^	0.03 ± 0.01^b^	0.24 ± 0.14^b^	5.01 ± 1.08^A,d,e^
C-peptide urine (nmol L^−1^)	19.0 ± 3.2	21.2 ± 4.3	19.1 ± 3.0	20.2 ± 4.5	26.6 ± 10.1	26.9 ± 5.9^A^
C-peptide/creatinine ratio urine	3.8 ± 0.3	3.8 ± 1.0	3.4 ± 0.4	2.7 ± 0.2	2.4 ± 0.5	4.0 ± 0.5^A^

Anthropometric data are means ± SD, with *n* or *n*(%); data of OMM and urinary glucose and C-peptide concentrations are means ± SEM. Symbols represent significance with *P* < 0.0125 in the corresponding subgroup versus other subgroups as stated in methods section

^a^Versus SA T2D

^b^Versus Cau T2D

^c^Versus SA NGT

^d^Versus Cau NGT

^e^Versus Cau IFG/IGT

^f^Versus SA IFG/IGT

^A^Caucasian T2D *n* = 11, for reasons mentioned in methods section

### Effect of renal glucose loss on plasma-derived OMM indices

The absolute amount of glucose concentration collected in urine voids of T2D patients did not influence glucose minimal model measurements, as a result of the small variation of renal clearance (range 0–2.1 %, with median 0.2 % of total plasma glucose AUC).

### Relation urine markers with plasma indices of C-peptide and glucose, OMM, and eGFR

Relationships between urinary glucose and C-peptide with plasma indices in both ethnicities can be found in Table 2. In both ethnicities, urinary glucose was positively associated with plasma glucose AUC and negatively associated with *S*_I_ and DI values. In both ethnicities, urinary C-peptide was positively associated with plasma C-peptide AUC. Plasma AUC of OMM flux *k*_01_ reflecting C-peptide from central compartment that is irreversibly metabolized, correlated with creatinine-adjusted urinary C-peptide (Fig. 2; *r* = 0.42; *P* < 0.01). Creatinine-unadjusted and adjusted urinary glucose as well as C-peptide had no significant correlation with eGFR.

**Table 2:** Spearman correlation between urine markers and plasma indices

Urine markers	Plasma indices						
	Incr C-peptide AUC	Incr. glucose AUC	*S*_I_	*Φ*_dynamic_	*Φ*_static_	DI_dynamic_	DI_static_
South Asians							
Glucose urine	−0.023	0.757**	−0.459**	−0.578**	−0.774**	−0.687**	−0.730**
Glucose/creatinine ratio urine	−0.059	0.690**	−0.414**	−0.565**	−0.727**	−0.653**	−0.674**
C-peptide urine	0.331*	0.170	−0.151	0.153	0.036	0.010	−0.060
C-peptide/creatinine ratio urine	0.301*	0.069	−0.148	0.100	0.068	−0.057	−0.002
Caucasians							
Glucose urine	0.175	0.669**	−0.639**	−0.208	−0.571**	−0.663**	−0.763**
Glucose/creatinine ratio urine	0.036	0.644**	−0.513**	−0.412**	−0.610**	−0.687**	−0.656**
C-peptide urine	0.485**	0.346*	−0.458**	0.178	−0.039	−0.269	−0.398**
C-peptide/creatinine ratio urine	0.453**	0.254	−0.202	−0.055	−0.053	−0.198	−0.168

* *P* < 0.05, ** *P* < 0.01

### Estimation of glucose threshold among non-T2D and T2D subgroups

Our stepwise exclusion approach to estimate glucose threshold during OGTT can be found in Table 3. It led to glucose threshold of ~10 mmol L^−1^ among Caucasian NGT and IFG/IGT individuals. In South Asians, glucose threshold for NGT and IFG/IGT individuals did not differ much, being ~10.5 mmol L^−1^. Glucose threshold during OGTT varied considerably among T2D patients from both ethnicities and was therefore not assessable.

**Table 3:** Two groups, one with and one without glucose in urine

	Caucasians^A^		South Asians	
	*N* (stepwise)	Glucose AUC	*N* (stepwise)	Glucose AUC
Threshold 8.5 mmol L^−1^				
Urine glucose−				
Non-T2D	3	28.8 ± 14.7	2	71.25 ± 57.0
T2D	0	0	1	33.8
Urine glucose+				
Non-T2D	19	116.6 ± 29.3	14	116.2 ± 28.9
T2D	11	1012.9 ± 143.4	20	684.5 ± 107.0
Threshold 9 mmol L^−1^				
Urine glucose−				
Non-T2D	2	17.25 ± 6.0	2	41.3 ± 37.5
T2D	0	0	1	12.0
Urine glucose+				
Non-T2D	16	103.6 ± 28.0	12	99.7 ± 26.0
T2D	11	922.7 ± 139.1	20	614.0 ± 102.2
Threshold 9.5 mmol L^−1^				
Urine glucose−				
Non-T2D	2	2.25 ± 1.50	1	45.8
T2D	0	0	0	0
Urine glucose+				
Non-T2D	11	112.1 ± 28.5	11	75.8 ± 23.0
T2D	11	837.8 ± 133.8	20	545.1 ± 97.3
Threshold 10 mmol L^−1^				
Urine glucose−				
Non-T2D	0	0	1	16.5
T2D	0	0	0	0
Urine glucose+				
Non-T2D	9	99.0 ± 25.6	8	67.5 ± 22.4
T2D	11	759.3 ± 128.5	20	480.3 ± 91.8
Threshold 10.5 mmol L^−1^				
Urine glucose−				
Non-T2D	0	0	0	0
T2D	0	0	0	0
Urine glucose+				
Non-T2D	8	77.6 ± 20.6	7	46.9 ± 18.6
T2D	11	683.1 ± 123.2	20	420.5 ± 85.8
Threshold 11.0 mmol L^−1^				
Urine glucose−				
Non-T2D	0	0	0	0
T2D	0	0	0	0
Urine glucose+				
Non-T2D	8	53.3 ± 16.5	7	25.9 ± 13.9
T2D	11	608.8 ± 117.8	19	383.8 ± 81.4
Threshold 11.5 mmol L^−1^				
Urine glucose−				
Non-T2D	0	0	0	0
T2D	0	0	0	0
Urine glucose+				
Non-T2D	7	38.6 ± 13.4	4	21.2 ± 13.0
T2D	11	538.9 ± 111.8	17	367.0 ± 78.7

With increasing glucose threshold, stepwise exclusion was performed separately among non-T2D (NGT + IFG/IGT) and T2D subgroups, based on their absence of having above threshold glucose AUC (mean ± SEM)

^A^Caucasian T2D *n* = 11, for reasons mentioned in methods section

### ROC values of urine markers for detection of glucose tolerance state

**Fig. 3:**
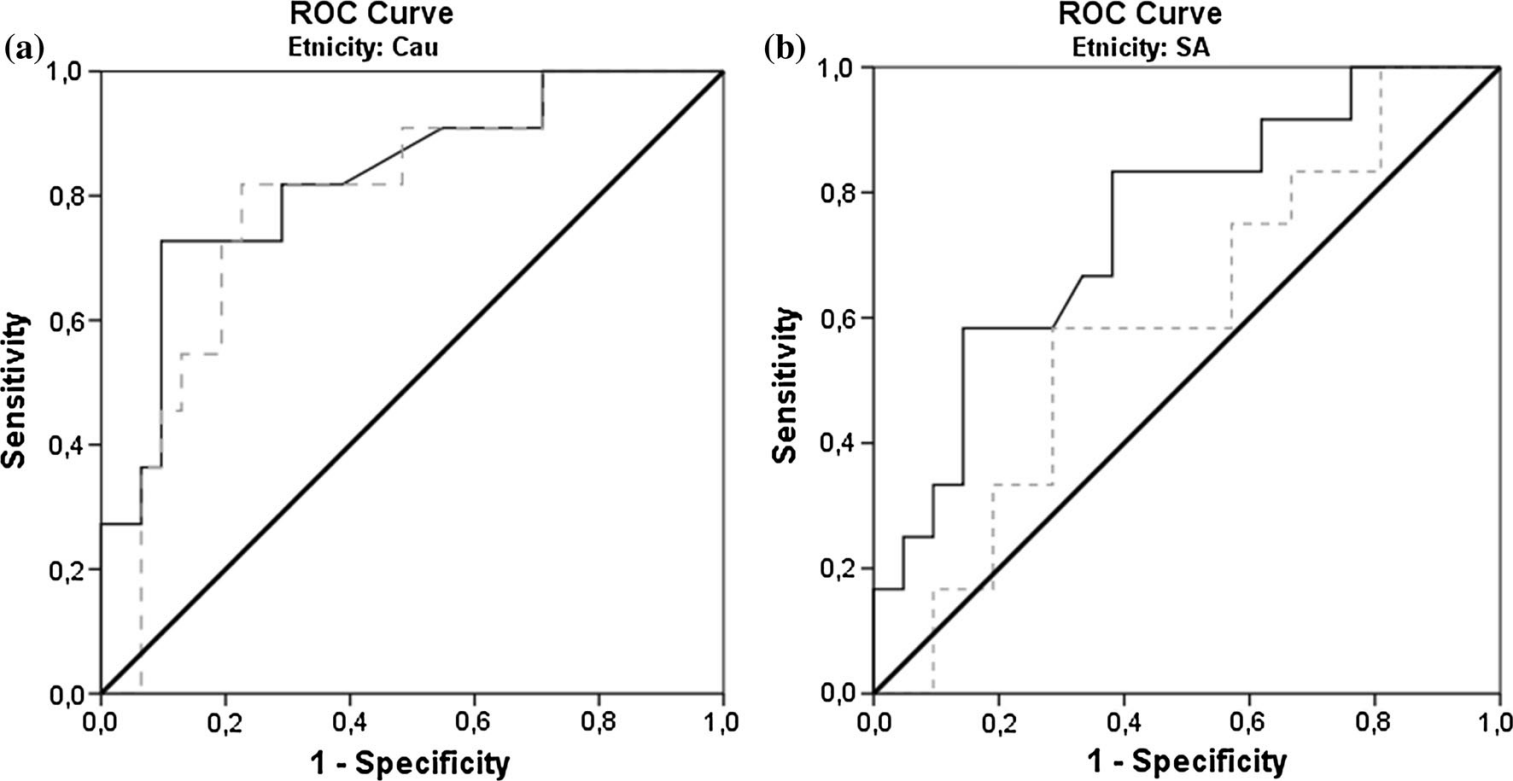
**a**, **b** Receiver-operated characteristics (ROC) curve for discriminatory ability between individuals with normal glucose tolerance (NGT) versus impaired fasting glucose/impaired glucose tolerance (IFG/IGT) with urinary glucose (*closed line*) or C-peptide (*dashed line*) concentration obtained from urine collected during OGTT (both unadjusted for urine creatinine), in Caucasians (**a**) and South Asians (**b**)

We examined the areas under ROC curves of glucose and C-peptide in urine unadjusted as well as adjusted for creatinine (adjusted are the values between brackets), respectively. We have found clear differences between the two ethnicities and therefore performed separate analyses. Urinary glucose concentration demonstrated high capability to discriminate between T2D and the combination of the other two subgroups (NGT and IFG/IGT), 0.976 (0.996) in Caucasians and 0.893 (0.898) in South Asians, respectively. We also calculated c-indices of urinary glucose for NGT versus the combination of IFG/IGT and T2D, 0.904 (0.908) in Caucasians and 0.894 (0.877) in South Asians, and c-indices for NGT versus the IFG/IGT subgroup, 0.826 (0.827) in Caucasians and 0.748 (0.736) in South Asians, respectively (Fig. 3).

The c-indices of urinary C-peptide concentration for the detection of glucose tolerance status were for discrimination between T2D and the combination of two other subgroups, 0.658 (0.742) in Caucasians and 0.503 (0.565) in South Asians, respectively. The c-indices for NGT versus the combination of IFG/IGT and T2D were 0.692 (0.733) in Caucasians and 0.556 (0.584) in South Asians. The c-indices for NGT versus IFG/IGT were 0.792 (0.792) in Caucasians and 0.595 (0.599) in South Asians, respectively (Fig. 3).

## Discussion

We found that urinary C-peptide collected during OGTT correlated with plasma C-peptide indices and OMM-derived estimates of renal C-peptide excretion. Urinary glucose collected during OGTT also correlated well with plasma indices, especially with plasma glucose AUC. Urinary glucose was mainly present in urine of patients with T2D; however, the loss of glucose in the urine was too small to influence general OMM calculations. Among the patients with T2D, the urinary glucose concentration showed large variation but discriminated well between normal and abnormal glucose tolerance.

Both glucose minimal model and C-peptide minimal model assess plasma glucose concentrations during OGTT with the first model also applying an area under the curve constraint; the amount of circulating glucose is expected to be a fixed parameter based on the amount of glucose load used as stimulus. The knowledge about the effects of dynamic glucose loss on OMM-based parameters due to renal handling after stimulus is limited [7]. We hypothesized that the variance of the urinary glucose excretion could be a serious confounder, but we found that renal loss of glucose does not influence OMM-based parameters of insulin sensitivity and beta-cell function. This finding is highly relevant for glucose homeostasis based kinetic models in general.

Overnight, 24-h fasting, and after a mixed meal, urine collection studies demonstrated the value of urinary C-peptide as non-invasive measure for endogenous insulin secretion in people with and without diabetes [12–17]. As stricter metabolic control affects urinary C-peptide, it might be of use to follow-up the insulin secretory function [18–20]. In line, we found that during OGTT, urinary C-peptide correlated well with plasma values reflecting the endogenous pancreatic secretion. For urinary C-peptide, we did not observe a relationship between eGFR MDRD. This is in agreement with previous studies, where the presence of micro albuminuria or renal impairment with reduced filtration rate did not alter the relationship between urinary and plasma C-peptide [21, 22].

Glucose is cleared by the kidney and predominantly reabsorbed by the sodium-glucose co-transporter 2 (SGLT2) in the proximal tubules. The urinary glucose excretion threshold is believed to be around 10 mmol L^−1^ in individuals without T2D [23, 24], which is in accordance with our estimations, with the exception of our individuals with T2D. Our patients with T2D did not use SGLT2 inhibitors. With clamp steady-state studies, it was demonstrated that a glucose threshold is not applicable to individuals with T2D due to a large variation in their glucose excretion; and glucosuria is present even when treated patients with T2D return to euglycemic conditions [25, 26]. Hence, using the OGTT the post-glucose-load urinary glucose concentration may be useful as non-invasive marker to detect abnormal glucose tolerance, but it is not suited to monitor treatment.

The strength of the present study lies in the fact that we used OMM and assessed urine parameters in two different ethnicities and in all stages of glucose tolerance. Among the weaknesses of our study are the limited sample size, the limited possibilities to translate our findings of the *extended* OGTT into clinical applications and using estimate eGFR MDRD instead of measuring GFR directly as a measure for renal function. Although our groups were relatively small, differences between subgroups and ethnicities became apparent with this relatively simple and low-cost test procedure. In contrast to the customary 24-h urine collections obtained at home, we collected urine in the hospital setting, during an extended version of OGTT. Validity of reduced amount of sampling and sampling time after stimulus has been demonstrated previously in healthy individuals, resulting in a more practical application of OMM [27]. We were also able to reduce amount of sampling, as we found no significant differences between plasma indices obtained from our above-described final 210 min-post-glucose load nine samples OGTT versus an earlier performed pilot with 210 min-post-glucose load 13 samples OGTT, which also included sampling at *t* = 5, *t* = 10, *t* = 20 and *t* = 25 min (data not shown). The participants in our study had no severe kidney failure and no history of renal disease.

In conclusion, urinary C-peptide corresponded well to OMM-derived estimates of renal C-peptide clearance and the renal glucose secretion during OGTT did not influence OMM indices.

## Abbreviations

AUC: Area under curve
DI: Disposition Index
eGFR: Estimated glomerular filtration rate
IS: Insulin sensitivity
MDRD: Modification of diet in renal disease
OMM: Oral minimal model
ROC: Receiver-operated characteristics
